# Bedside sonography to diagnose bladder trauma in the emergency department

**DOI:** 10.4103/0974-2700.66529

**Published:** 2010

**Authors:** Tanweer Karim, Margaret Topno

**Affiliations:** Department of Surgery, M.G.M’s Medical, College & Hospital, Kamothe, Navi Mumbai, India

Sir,

Urinary tract injuries occur in 10–15% of blunt and penetrating abdominal trauma,[1] and 15% of all pelvic fractures are associated with concomitant bladder or urethral injuries.[2] Bladder trauma is seldom an isolated injury and is frequently missed in poly-trauma cases because of attention to more life-threatening injuries like head injury, tension pneumothorax, hemothorax and hemoperitoneum.[3]

The methods of diagnosis and management of such injuries are well established and accepted. Abdominal computed tomography (CT) is inferior to the retrograde cystogram as a method of detecting bladder injury, unless cystography is used as an adjunct to CT. CT abdomen alone has an accuracy of 50–60% only, whereas conventional retrograde cystogram is 95–100% accurate in diagnosing bladder rupture.[4,5] However, it is difficult to perform conventional retrograde cystogram and CT cystogram in a critically ill patient because the average time required is more than 30 minutes. Moreover, they cannot be performed bedside, leading to unwanted delay.

With the advent of high-frequency probes, evaluation of urinary bladder and free fluid in pelvis can be done with ultrasonography with a fair accuracy when bladder is distended. Detection of peritoneal fluid in the presence of normal viscera or failure to visualize the bladder after the transurethral introduction of saline is considered highly suggestive of bladder rupture.[6]

We used retrograde instillation of saline because patients of abdominal trauma are routinely subjected to bedside ultrasonography in our setting. The addition of this technique at the same place will help us in diagnosing bladder injuries without much delay and adding no cost. Patients with suspected bladder injury were first subjected to retrograde instillation of sterile normal saline through Foley’s catheter and later viewed on ultrasonography for the presence or absence of distension of bladder and disruption of bladder wall. Those patients with obvious urethral injuries were not included.

Retrospectively, we found that out of 103 patients of polytrauma admitted during 1 year from May 2005 to May 2006, 22 patients were having clinical features suggestive of bladder rupture. Ultrasonography with retrograde saline instillation was positive for bladder rupture in 20 patients. Subsequently, all the 22 patients were operated for possible bladder rupture. Eighteen of them had extra-peritoneal rupture and four had intra-peritoneal rupture. False negative tests were seen in two cases of extra-peritoneal rupture.

Therefore, in conditions when accurate and rapid disposition of polytrauma patients is crucial for optimal outcome, saline instillation under Ultrasonography can help in making early diagnosis of bladder rupture relatively simple and easy with fair accuracy (sensitivity = 90%, positive predictive value = 1). Although accuracy (sensitivity and specificity) of bedside ultrasonography needs further evaluation in a larger sample size, this appears to have the following advantages over retrograde cystogram and CT cystogram:

1. it is simple and rapidly performed (average time 8 minutes);
2. it can be performed bedside in critically ill patients;
3. no risk of radiation exposure is associated with CT cystogram; and
4. no risk of anaphylaxis is associated with contrast material.

## References

[1] Corriere JN, Sandler CM (1988). Mechanisms of injury, patterns of extravasation and management of extraperitoneal bladder rupture due to blunt trauma. J Urol.

[2] Hochberg E, Stone NN (1993). Bladder rupture associated with pelvic fracture due to blunt trauma. Urology.

[3] Ziran BH, Chamberlin E, Shuler FD, Shah M (2005). Delays and difficulties in the diagnosis of lower urologic injuries in the context of pelvic fractures. J Trauma.

[4] Horstman WG, McClennan BL, Heiken JP (1991). Comparison of computed tomography and conventional cystography for detection of traumatic bladder rupture. Urol Radiol.

[5] Haas CA, Brown SL, Spirnak JP (1999). Limitations of routine spiral computerized tomography in the evaluation of bladder trauma. J Urol.

[6] Sandler CM, Francis IR, Baumgarten DA, Bluth EI, Bush WH, Casalino DD (2007). Expert Panel on Urologic Imaging; Suspected lower urinary tract trauma.

